# Psychological trajectories following brief mindfulness training: a longitudinal comparison of active and passive controls

**DOI:** 10.3389/fpsyg.2026.1836930

**Published:** 2026-08-26

**Authors:** Weiqiang Qi, Xiaodong Li, Xinyue Liang, Maodan Shen, Yichun Zhuo, Weifu Tang, Yujia Xiang, Taiyong Bi, Hui Kou

**Affiliations:** 1 Research Center of Humanities and Medicine, Zunyi Medical University, Zunyi, China; 2 GZU-ZMU Joint Laboratory of Brain Intelligence Development and Applied Ethics, Zunyi, China

**Keywords:** anxiety and depression, emotional regulation, mindfulness training, psychological symptoms, randomized controlled trial

## Abstract

**Background:**

Mindfulness-based interventions (MBIs) have been demonstrated to enhance emotional functioning, but little is known regarding the brief mindfulness training’s effects on various psychological symptoms and positive psychological resources in the long term, especially when compared to both passive and active control conditions. This study aimed to examine the effects of brief mindfulness training on psychological symptoms compared with active and passive control conditions across three time points.

**Methods:**

This study was designed as three-wave longitudinal, three-arm randomized controlled trial. (*N* = 180) participants were randomly grouped into mindfulness training group (MG), breathing relaxation group (active control, ACG), and no-treatment control group (passive control, PCG). The psychological outcomes (anxiety, depression, state and trait mindfulness, attentional control, emotional intelligence, and psychological resilience) were determined at baseline (T1), post-intervention (T2), and 2 weeks follow-up (T3). Psychological outcomes were assessed using validated self-report instruments, including the Self-Rating Anxiety Scale (SAS), Self-Rating Depression Scale (SDS), State Mindfulness Scale (SMS), Five Facet Mindfulness Questionnaire–Short Form (FFMQ-SF), Attentional Control Scale (ACS), Emotional Intelligence Scale (EIS), and Connor–Davidson Resilience Scale (CD-RISC). Repeated-measures ANOVAs were used to test Group × Time effects.

**Results:**

There were no significant differences in baseline between groups. There was a significant Group × Time interaction in all the measured outcomes. The mindfulness group (MG) compared to both control groups had greater post-intervention anxiety and depression reductions and state and trait mindfulness, attentional control, emotional intelligence, and resilience increases. The ACG showed slight within-group changes but not better than the PCG.

**Conclusion:**

Brief mindfulness training had specific, time-varying changes in psychological symptoms and positive psychological resources. These results suggest that mindfulness has more than a general relaxation or engagement effect, which demonstrates its effectiveness as an intervention that can redefine the psychological functioning by using dynamic, targeted attentional regulation processes.

## 1 Introduction

Depression and anxiety are examples of mental health problems that affect university students, which are conditioned by academic stress and social pressure (Drescher et al., 2023; González-Martín et al., 2023). Globally, a meta-analysis reported that approximately 33.6% of college students experienced depressive symptoms and 39.0% experienced anxiety symptoms, highlighting the need for accessible psychological interventions in this population (Li et al., 2022). Although the longer-term effects of mindfulness interventions have been examined, including follow-up periods of up to 6 months, the magnitude and consistency of these effects vary across the outcomes assessed (Goyal et al., 2014). MBIs (Mindfulness-based interventions) have been demonstrated to decrease anxiety, depression, and stress (Alrashdi et al., 2024; Zuo et al., 2023). Nevertheless, the results of different studies are inconsistent, and a large number of them are based on PCGs (Passive Control Groups), which complicates the separation of the peculiarities of mindfulness (Alrashdi et al., 2024). The longevity of these effects remains ambiguous as well since few follow-ups and high dropout rates in online or self-managed programs have been used (Zinke et al., 2025). Moreover, most studies consider single outcomes without considering the time sensitivity of changes that play a vital role in comprehending the overall effect of MBIs (Galante et al., 2023; O’Connor et al., 2023). The absence of ACGs (Active Control Groups) in previous trials also restricts the possibility of separating mindfulness-specific and nonspecific effects of mindfulness meditation that include participant engagement or expectancy (Lin et al., 2025; Remskar et al., 2024). Passive control groups (PCGs) receive no intervention and provide a reference for changes related to time, repeated assessment, or natural fluctuations. Active control groups (ACGs), in contrast, receive a structurally comparable activity and help control for nonspecific factors such as researcher contact, session duration, engagement, and relaxation. Therefore, comparisons with both control groups allow a more specific evaluation of whether the observed changes are associated with mindfulness-related components rather than with participation or the passage of time alone. Recent meta-analyses of mindfulness interventions delivered via apps suggest only small benefits as compared to controls and no significant benefit compared to active therapeutic interventions, which indicates the necessity of more rigorous study designs (Linardon et al., 2024).

Mindfulness is a deliberate act of directing attention and mindfulness to the current internal and external experiences through curiosity, openness, receptiveness, and non-judgmental attitude (Allen et al., 2006; Kabat-Zinn, 2023). In this context, the benefits of mental health are believed to be achieved mainly in terms of improved attentional control, which, in turn, contributes to emotion-related abilities. Recent randomized studies have started to elucidate the attentional mechanisms connecting mindfulness training with symptom reduction, and results have been found to support attentional control as a mediator of stress reduction in controlled studies (Mora Álvarez et al., 2023)(Cásedas et al., 2022; Mitsea et al., 2023; Roca et al., 2023; Wang et al., 2024).

Mindfulness is a supported clinical intervention. Randomized trials suggest that mindfulness meditation is able to reach clinically significant results, just as first-line pharmacotherapy of anxiety disorders (Hu et al., 2024). Nevertheless, issues of its use in non-clinical populations and in scalable formats still exist. Short, low-intensity interventions are especially attractive in the university environment, but they need to be thoroughly compared with plausible comparisons (Remskar et al., 2024). In addition to the reduction of symptoms, mindfulness can also increase more global psychological resources. According to meta-analytic findings, mindfulness-based interventions (MBIs) have the potential to enhance resilience and may have more significant benefits than active controls in the long run, but not in the short term, which underscores the role of longitudinal designs (O’Connor et al., 2023). Moreover, mindfulness is often theorized as a core competency that facilitates emotion-related skills, including emotional awareness, emotion regulation, and interpersonal functioning. According to the recent controlled research in the educational setting, mindfulness practice may enhance emotional and attention-related outcomes, but the extent and sustainability of these effects depend on the specifics of the population and the program (Lin et al., 2025).

The current research provides important methodological and substantive gaps in the literature on mindfulness by using a three-arm randomized controlled trial with an active and passive control, repeated measurements of the outcomes with time, and a multidimensional outcome measure that included symptoms and psychological abilities. Precisely, we compared a short meditation session with relaxation using breathing and no-intervention control at three points: baseline (T1), postintervention (T2), and two-week follow-up (T3). Some of the outcomes included state mindfulness, trait mindfulness, anxiety, depression, attentional control, emotional intelligence, mindfulness awareness, and psychological resilience. Drawing on recent integrative models of mindfulness-related cognitive and emotional change, the seven outcomes were organized along a proximal-to-distal pathway (Cásedas et al., 2024; Raugh et al., 2025). Brief mindfulness practice was expected to first influence state mindfulness and attentional control, which may subsequently affect emotion regulation and emotional symptoms such as anxiety and depression. Trait mindfulness, emotional intelligence, and resilience were included as broader and relatively stable psychological resources.

To provide a more rigorous assessment of mindfulness-related changes, we incorporated a three-arm design with active and passive control conditions, repeated assessments, and multiple psychological outcomes. To begin with, it separates mindfulness-specific effects as opposed to general relaxation or slow breathing. Second, it explores the issue of whether the impact of mindfulness is specific or broader. Third, it examines whether the immediate changes associated with a single 15 min mindfulness session remain detectable at a two-week short-term follow-up. Accordingly, we hypothesized that: (H1) compared with the ACG and PCG, the MG would show greater increases in state mindfulness, attentional control, trait mindfulness, emotional intelligence, and resilience, together with reductions in anxiety and depressive symptoms from T1 to T2; and (H2) these improvements would partially diminish at the two-week follow-up in the absence of continued mindfulness practice (Galante et al., 2023; O’Connor et al., 2023; Wang et al., 2024).

## 2 Materials and methods

### 2.1 Participants and procedure

A total of 180 undergraduate students were recruited via online advertisements and randomly allocated to 1 of 3 groups: mindfulness training (MG; *n* = 60) or breathing relaxation (active control, ACG; *n* = 60) or no-intervention control (passive control, PCG; *n* = 60). The random allocation sequence was generated using a computer-based random-number generator, with participants assigned to the three groups in a 1:1:1 ratio. Participants were between 18 and 23 years old (*M* = 19.81, SD = 1.15), and group means were 19.53 years (SD = 1.24) for the MG, 20.00 years (SD = 0.94) for the ACG, and 19.90 years (SD = 1.20) for the PCG, with no significant differences between groups (*F*(2, 177) = 2.81, *p* = 0.063 Gender distribution was the same in all groups (15 males and 45 females each, male:female ratio = 1:3). During recruitment, all participants reported no prior formal mindfulness or meditation experience.

This study was designed as a single-blind, three-wave longitudinal, three-arm randomized controlled trial. Participants were not informed of the specific study hypotheses or their assigned intervention condition. Assessments were conducted at baseline (T1), post-intervention (T2), and two-week follow-up (T3). At T1, participants completed a battery of online self-report measures, including the Emotional Intelligence Scale (EIS), Connor-Davidson Resilience Scale (CD-RISC), State Mindfulness Scale (SMS), Five Facet Mindfulness Questionnaire-Short Form (FFMQ-SF), Self-Rating Anxiety Scale (SAS), Self-Rating Depression Scale (SDS), and Attentional Control Scale (ACS). After the baseline assessment, participants were randomly assigned to the mindfulness training group (MG), active control group (ACG), or passive control group (PCG).

On the following day, participants in the MG and ACG attended one 15 min in-person session. In the MG, the session was delivered by a trained researcher using a standardized script and focused on breath awareness, present-moment bodily sensations, monitoring mind wandering, and returning attention with an open and non-judgmental attitude. In the ACG, the session was matched with the MG in duration, delivery format, and researcher contact, but focused only on slow breathing and physical relaxation, without mindfulness-specific elements such as open monitoring, non-judgmental awareness, or observation of thoughts and emotions. Participants in the PCG received no intervention and were asked to maintain their usual daily activities.

Immediately after the MG and ACG sessions on the second day, participants in all three groups completed the same questionnaire battery at T2. Because both the SAS and SDS assess symptoms experienced during the preceding week, the retrospective periods at T1 and the immediate post-intervention T2 assessment substantially overlapped. These measures were retained at T2 to maintain consistency across assessment waves; however, immediate T1-T2 differences were interpreted as possible changes in participants’ self-reported affective state or symptom appraisal rather than as clear changes in past-week anxiety or depressive symptoms. During the two-week interval between T2 and T3, no further intervention was provided to any group. At T3, all participants completed the same assessments to evaluate the maintenance of intervention effects.

### 2.2 Ethics approval statement

All procedures including human participants were performed per ethical standards of the Ethical Committee of Human Research at Zunyi Medical University and the 1964 Helsinki Declaration, including its later amendments and comparable ethical standards. Informed consent was sourced from all participants before participating in the study. After completion of the research, participants in the control groups were given access to the mindfulness training program if they requested it.

### 2.3 Measures

#### 2.3.1 Attentional Control Scale (ACS)

Attentional control was determined by the ACS of Chinese version (Derryberry and Reed, 2002; Li et al., 2017). The ACS is a 20-item self-report measure on a 5-point Likert scale, 7 items are reverse-scored. Total scores are calculated by adding up all of the items (range: 20–100) and higher scores reflect greater attentional control. In the current study, the ACS showed an excellent internal consistency (Cronbach’s *α* = 0.872) and sampling adequacy (KMO = 0.864).

#### 2.3.2 Emotional Intelligence Scale (EIS)

Emotional intelligence was measured using the Chinese version (Shi and Wang, 2007; Wong and Law, 2017). The EIS contains 16 items in four dimensions: self-emotion appraisal (items 1–4), other’s emotion appraisal (items 5–8), regulation of emotion (items 9–12), and use of emotion (items 13–16). Items are scored on a 7-point scale (1 = strongly disagree, 7 = strongly agree) and average scores are calculated, with higher scores indicating greater emotional intelligence. In this study, the EIS was revealed to have good internal consistency (Cronbach’s *α* = 0.855) and sampling adequacy (KMO = 0.802).

#### 2.3.3 Connor–Davidson Resilience Scale (CD-RISC)

Psychological resilience was measured by the CD-RISC (Connor and Davidson, 2003). The Chinese version (Xiaonan and Jianxin, 2007) contains 25 items covering 3 dimensions: resilience, strength and optimism. Moreover, items are rated on a 5-point scale (0 = not true at all, 4 = true nearly all the time) with total scores calculated such that higher scores reflect greater resilience. In this study, the CD-RISC was illustrated to have good internal consistency (Cronbach’s *α* = 0.903) and sampling adequacy (KMO = 0.887).

#### 2.3.4 Self-Rating Anxiety Scale (SAS)

Anxiety symptoms were measured using the Chinese version of the Self-Rating Anxiety Scale (Wang et al., 1999; Zung, 1971a,b). The SAS is comprised of 20 items that are rated on a 4-point scale according to the experiences of the participants over the past week. Raw scores (range: 20–80) were summed and converted to standard scores using the formula 1.25 multiplied by the raw score and rounding (range: 25–100), with higher scores signifying greater anxiety. In this study, the SAS showed an exceptional internal consistency (Cronbach’s *α* = 0.851) and sampling adequacy (KMO = 0.854).

#### 2.3.5 Self-Rating Depression Scale (SDS)

Depressive symptoms were measured using the Chinese version of the Self-Rating Depression Scale (Wang et al., 1999; Zung, 1965). The SDS is a 20-item rating scale on a 4-point scale of experiences over the past week. Raw scores (range: 20–80) were summed and transformed into standard scores by multiplying the sum by 1.25 and rounding (range: 25–100), where higher scores are associated with more severe depressive symptoms. In the present study, the SDS was found to have good internal consistency (Cronbach’s *α* = 0.785) and sampling adequacy (KMO = 0.817).

#### 2.3.6 Five Facet Mindfulness Questionnaire–Short Form (FFMQ-SF)

Trait mindfulness was determined with the short form of the FFMQ-SF, adapted and validated for the Chinese sample (Meng et al., 2019). This 20-item version measures five facets: describing, observing, acting with awareness, non-reactivity, and non-judging. Participants rated items on a Likert scale according to the scoring instructions of the adapted version. Total scores were obtained by adding up all the items, where the higher the score, the more trait mindfulness was perceived. In this study, the FFMQ-SF indicated acceptable internal consistency (Cronbach’s *α* = 0.768) and sampling adequacy (KMO = 0.800).

#### 2.3.7 State Mindfulness Scale (SMS)

State mindfulness was measured with the Chinese version of the SMS (Tanay and Bernstein, 2013; Xiaoshuo et al., 2023), a 21-item self-report measure with two dimensions of mindfulness of body and mindfulness of mind. Participants rated items according to their current experience using a Likert scale and total scores were determined with higher scores representing higher state mindfulness. In this study, the SMS showed great internal consistency (Cronbach’s *α* = 0.925) and sampling adequacy (KMO = 0.911).

### 2.4 Data analysis

To test the differences in the baseline (T1) groups on all variables, first, one-way analyses of difference (ANOVAs) were utilized to test the differences. Second, a 3 (Group: MG vs. ACG vs. PCG) × 3 (Time: T1 vs. T2 vs. T3) repeated-measures ANOVA was conducted on each outcome to test the Group × Time interaction and to test whether the change trajectories of the interventions were different. In cases where the Group × Time interaction was significant, simple effects analyses were followed up at the follow-up to investigate within-group and between-group differences across time. Multiple comparisons were addressed at two levels. The seven primary Group × Time interaction tests were corrected using the Benjamini–Hochberg false discovery rate (FDR) procedure. For significant interactions, post-hoc pairwise comparisons were further adjusted using Bonferroni correction within each outcome. Statistical significance was set at *p* < 0.05 after correction.

## 3 Results

### 3.1 Baseline comparison

As shown in Table 1, one-way ANOVAs indicated no significant baseline (T1) differences between the three groups on the SMS, FFMQ-SF, SAS, SDS, ACS, EIS or CD-RISC (all *p* > 0.05) suggesting that the three groups were similar before the intervention. Descriptive statistics for all outcome measures across the three groups at T1, T2, and T3 are presented in Table 2.

**Table 1:** The baseline level of variables across groups (*M* ± SD).

Variable	ACG (*n* = 60)	MG (*n* = 60)	PCG (*n* = 60)	*F* (2, 177)	*p*
SMS (T1)	3.71 ± 0.51	3.62 ± 0.57	3.58 ± 0.56	0.784	0.458
FFMQ-SF (T1)	85.23 ± 11.47	86.75 ± 11.80	85.45 ± 13.14	0.273	0.761
SAS (T1)	45.69 ± 9.36	43.31 ± 8.87	44.71 ± 9.42	1.006	0.368
SDS (T1)	50.21 ± 8.89	48.63 ± 8.62	49.54 ± 9.46	0.468	0.627
ACS (T1)	50.75 ± 7.85	49.83 ± 8.10	50.43 ± 7.24	0.217	0.805
EIS (T1)	5.05 ± 0.65	4.95 ± 0.60	4.93 ± 0.80	0.506	0.604
CD-RISC (T1)	3.43 ± 0.52	3.35 ± 0.47	3.40 ± 0.45	0.379	0.685

MG, mindfulness training group; ACG, active control group; PCG, passive control group; SMS, State Mindfulness Scale; FFMQ-SF, Five Facet Mindfulness Questionnaire–Short Form; SAS, Self-Rating Anxiety Scale; SDS, Self-Rating Depression Scale; ACS, Attentional Control Scale; EIS, Emotional Intelligence Scale; CD-RISC, Connor–Davidson Resilience Scale; T1, baseline.

**Table 2:** Means and standard deviations of outcome measures by group at each assessment point.

Outcome	Group	T1 (*M* ± SD)	T2 (*M* ± SD)	T3 (*M* ± SD)
SMS	MG	3.62 ± 0.57	4.19 ± 0.55	3.82 ± 0.54
	ACG	3.71 ± 0.51	3.72 ± 0.55	3.85 ± 0.55
	PCG	3.58 ± 0.56	3.63 ± 0.52	3.71 ± 0.57
FFMQ-SF	MG	86.75 ± 11.80	97.17 ± 16.10	88.63 ± 12.25
	ACG	85.23 ± 11.47	86.02 ± 11.97	86.10 ± 12.33
	PCG	85.45 ± 13.14	86.02 ± 13.50	87.18 ± 13.05
SAS	MG	43.31 ± 8.87	36.40 ± 7.52	42.60 ± 9.20
	ACG	45.69 ± 9.36	43.94 ± 9.65	46.71 ± 11.04
	PCG	44.71 ± 9.42	42.81 ± 8.39	44.17 ± 9.67
SDS	MG	48.63 ± 8.62	41.40 ± 8.02	47.19 ± 7.76
	ACG	50.21 ± 8.89	48.94 ± 9.62	50.81 ± 10.29
	PCG	49.54 ± 9.46	49.21 ± 9.61	49.29 ± 9.75
AC	MG	49.83 ± 8.10	57.90 ± 10.44	52.08 ± 7.75
	ACG	50.75 ± 7.85	53.20 ± 9.08	52.03 ± 9.46
	PCG	50.43 ± 7.24	51.00 ± 7.70	51.72 ± 8.32
EIS	MG	4.95 ± 0.60	5.53 ± 0.68	5.20 ± 0.65
	ACG	5.05 ± 0.65	5.15 ± 0.69	5.22 ± 0.72
	PCG	4.93 ± 0.80	4.96 ± 0.75	5.09 ± 0.73
CD-RISC	MG	3.35 ± 0.47	3.81 ± 0.48	3.59 ± 0.46
	ACG	3.43 ± 0.52	3.44 ± 0.56	3.51 ± 0.55
	PCG	3.40 ± 0.45	3.39 ± 0.55	3.43 ± 0.49

Values are presented as mean ± standard deviation. *n* = 60 per group. T1, baseline; T2, mid-intervention; T3, post-intervention; MG, mindfulness group; ACG, active control (breathing) group; PCG, passive control group.

### 3.2 State mindfulness (SMS)

**Figure 1:**
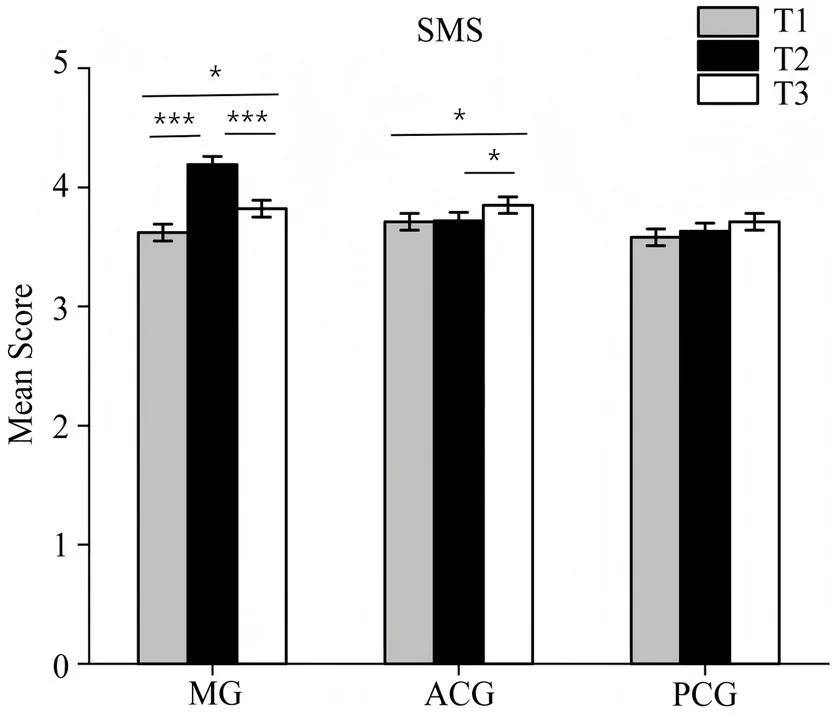
SMS scores for the MG, ACG), and PCG at T1 (pretest), T2 (posttest), and two-week T3 (follow-up). Error bars indicate standard errors of the mean (SEM). Significant within-group differences between time points are indicated by asterisks. *p < 0.05; **p < 0.01; ***p < 0.001, Bonferroni-corrected.

As shown in Figure 1, a significant Group × Time interaction was observed for state mindfulness, *F* (4, 354) = 17.982, *p* < 0.001, *η*^2^*_p_* = 0.169 (Figure 1). Simple effects analysis suggested that, in the MG, state mindfulness at T2 was significantly greater than state mindfulness at both T1 and T3 (*p* < 0.001, Bonferroni corrected). Scores at T3 were significantly higher than scores at T1 (*p* = 0.013, Bonferroni corrected). In the ACG, state mindfulness at T3 was significantly greater than state mindfulness at both T1 (*p* = 0.033, Bonferroni corrected) and T2 (*p* = 0.017, Bonferroni corrected). There was no significant difference in scores between T1 and T2 (*p* > 0.05, Bonferroni corrected). In the PCG, no significant differences in state mindfulness scores were observed between any of the time points (all *p* > 0.05, Bonferroni corrected).

### 3.3 Mindfulness awareness (FFMQ-SF)

**Figure 2:**
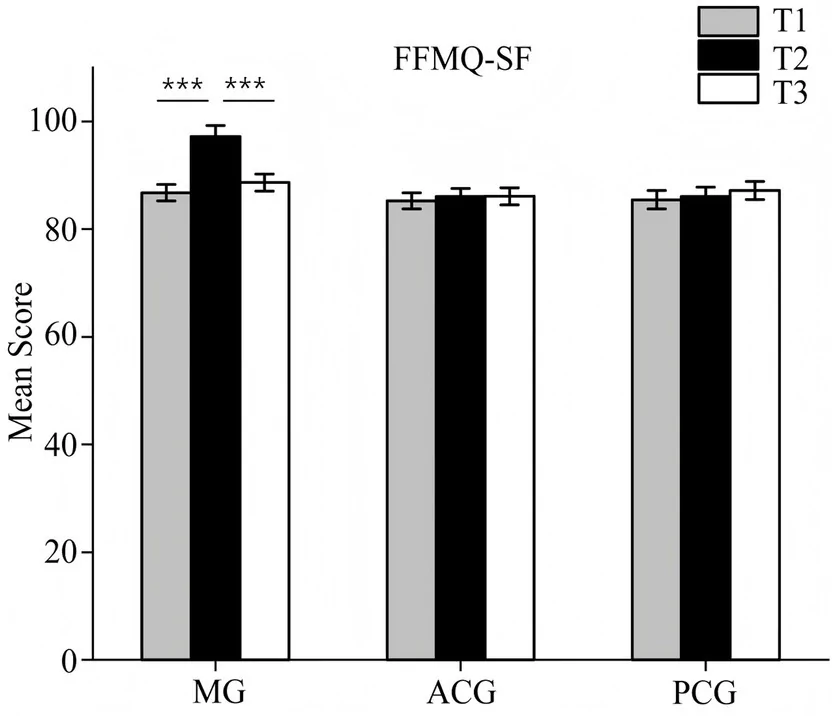
Five Facet Mindfulness Questionnaire–Short Form (FFMQ-SF) scores across groups and time points. Mean FFMQ-SF scores are presented for the MG, ACG, and PCG at T1 (pretest), T2 (posttest), and T3 (follow-up). Error bars represent SEM. Significant within-group differences between time points are indicated by asterisks. *p < 0.05; **p < 0.01; ***p < 0.001, onferroni-corrected.

As shown in Figure 2, a significant Group × Time interaction was observed for trait mindfulness, *F*(4, 354) = 13.45, *p* < 0.001, *η*^2^*_p_* = 0.132 (Figure 2). Simple effect analysis indicated that in the MG, the trait mindfulness score at T2 was significantly higher than those at T1 (*p* < 0.001, Bonferroni corrected) and T3 (*p* < 0.001, Bonferroni corrected). The score at T3 did not differ significantly from that at T1 (*p* > 0.05, Bonferroni corrected). In the ACG, no significant differences in trait mindfulness scores were observed between any of the time points (all *p* > 0.05, Bonferroni corrected). Similarly, in the PCG, no significant differences were found across the three time points (all *p* > 0.05, Bonferroni corrected).

### 3.4 Anxiety (SAS)

**Figure 3:**
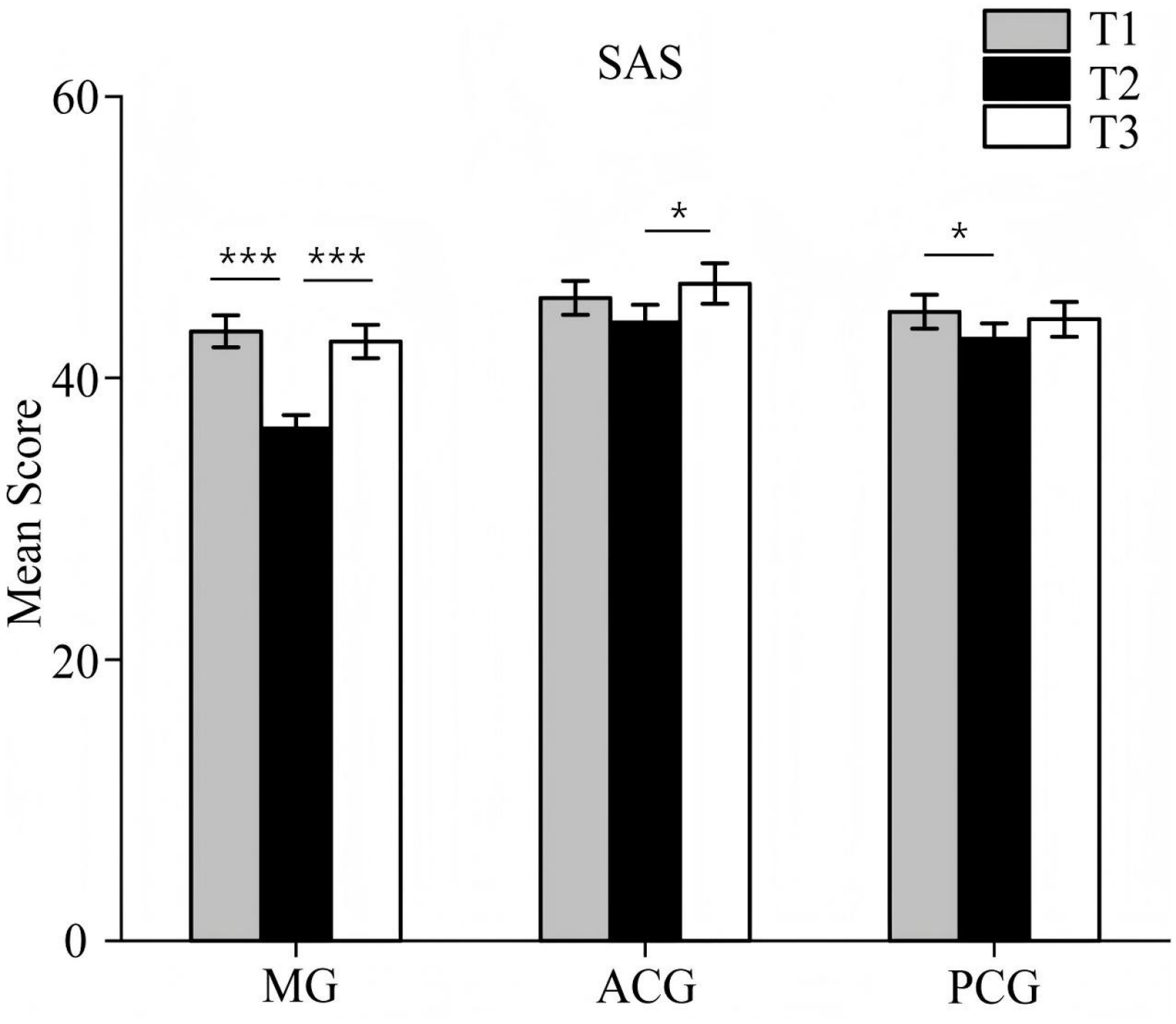
Self-Rating Anxiety Scale (SAS) scores across groups and time points. Mean SAS scores are presented for the MG, ACG, and PCG at T1 (pretest), T2 (posttest), and T3 (follow-up). Error bars represent SEM. Significant within-group differences between time points are indicated by asterisks. *p < 0.05; **p < 0.01; ***p < 0.001, Bonferroni-corrected.

As shown in Figure 3, a significant Group × Time interaction was observed for anxiety symptoms, *F*(4, 354) = 6.26, *p* < 0.001, *η*^2^*_p_* = 0.066 (Figure 3). Simple effect analysis indicated that in the MG, the anxiety score at T2 was significantly lower than those at T1 (*p* < 0.001, Bonferroni corrected) and T3 (*p* < 0.001, Bonferroni corrected). The score at T3 did not differ significantly from that at T1 (*p* > 0.05, Bonferroni corrected). In the ACG, the anxiety score at T2 did not differ significantly from that at T1 after Bonferroni correction (*p* = 0.050), but was significantly lower than that at T3 (*p* = 0.033, Bonferroni corrected), with no significant difference between T1 and T3 (*p* > 0.05, Bonferroni corrected). In the PCG, the anxiety score at T2 was significantly lower than that at T1 (*p* = 0.005, Bonferroni corrected), but no significant differences were observed between T2 and T3 (*p* > 0.05, Bonferroni corrected) or between T1 and T3 (*p* > 0.05, Bonferroni corrected).

### 3.5 Depression (SDS)

**Figure 4:**
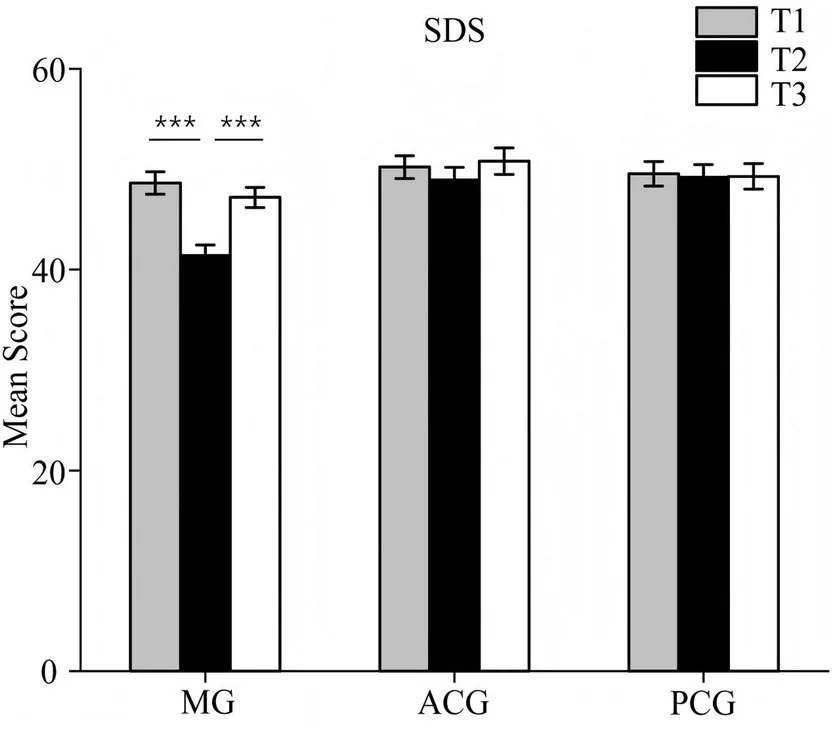
SDS scores across groups and time points. Mean SDS scores are presented for the MG, ACG, and PCG at T1 (pretest), T2 (posttest), and T3 (follow-up). Error bars represent standard errors of the mean (SEM). Significant within-group differences between time points are indicated by asterisks. *p < 0.05; **p < 0.01; ***p < 0.001, Bonferroni-corrected.

As shown in Figure 4, a significant Group × Time interaction was observed for depressive symptoms, *F* (4, 354) = 11.85, *p* < 0.001, *η*^2^*_p_* = 0.118 (Figure 4). Simple effect analysis indicated that in the MG, the depression score at T2 was significantly lower than those at T1 (*p* < 0.001, Bonferroni corrected) and T3 (*p* < 0.001, Bonferroni corrected). The score at T3 did not differ significantly from that at T1 (*p* > 0.05, Bonferroni corrected). In the ACG, no significant differences in depression scores were observed between any of the time points (all *p* > 0.05, Bonferroni corrected). Similarly, in the PCG, no significant differences were found across the three time points (all *p* > 0.05, Bonferroni corrected).

### 3.6 Attentional control (ACS)

**Figure 5:**
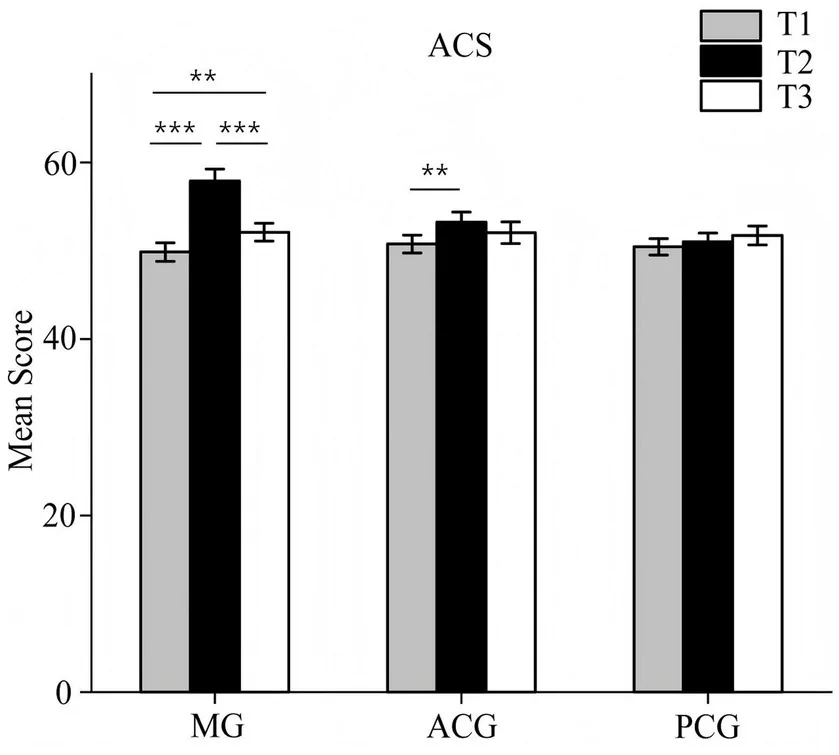
Attentional Control Scale (ACS) scores across groups and time points. Mean ACS scores are presented for MG, ACG, and PCG at T1 (pretest), T2 (posttest), and T3 (follow-up). Error bars represent SEM. Significant within-group differences between time points are indicated by asterisks. *p < 0.05; **p < 0.01; ***p < 0.001, Bonferroni-corrected.

As shown in Figure 5, a significant Group × Time interaction was observed for attentional control, *F* (4, 354) = 12.04, *p* < 0.001, *η*^2^*_p_* = 0.12 (Figure 5). Simple effect analysis indicated that in the MG, the attentional control score at T2 was significantly higher than those at T1 (*p* < 0.001, Bonferroni corrected) and T3 (*p* < 0.001, Bonferroni corrected). The score at T3 was significantly higher than that at T1 (*p* = 0.008, Bonferroni corrected). In the ACG, the attentional control score at T2 was significantly higher than that at T1 (*p* = 0.004, Bonferroni corrected), but no significant differences were observed between T2 and T3 (*p* > 0.05, Bonferroni corrected) or between T1 and T3 (*p* > 0.05, Bonferroni corrected). In the PCG, no significant differences in attentional control scores were observed between any of the time points (all *p* > 0.05, Bonferroni corrected).

### 3.7 Emotional intelligence (EIS)

**Figure 6:**
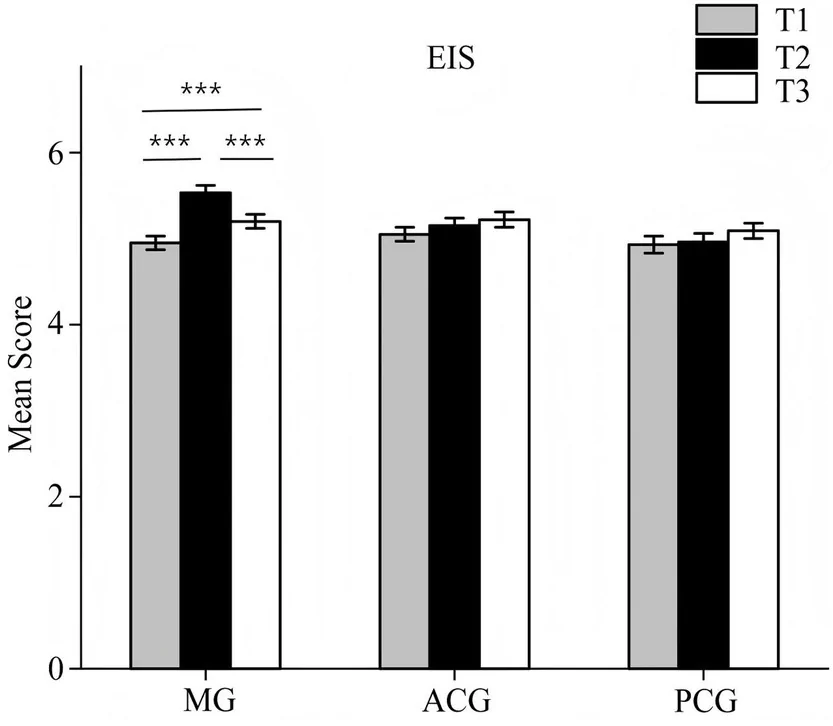
EIS scores across groups and time points. Mean EIS scores are presented for the MG, ACG, and PCG at T1 (pretest), T2 (posttest), and T3 (follow-up). Error bars represent standard errors of the mean (SEM). Significant within-group differences between time points are indicated by asterisks. *p < 0.05; **p < 0.01; ***p < 0.001, bonferroni-corrected.

As shown in Figure 6, a significant Group × Time interaction was observed for emotional intelligence, *F*(4, 354) = 9.582, *p* < 0.001, *η*^2^*_p_* = 0.098 (Figure 6). Simple effect analysis indicated that in the MG, the emotional intelligence score at T2 was significantly higher than those at T1 (*p* < 0.001, Bonferroni corrected) and T3 (*p* < 0.001, Bonferroni corrected). The score at T3 was significantly higher than that at T1 (*p* < 0.001, Bonferroni corrected). In the ACG, no significant differences in emotional intelligence scores were observed between any of the time points (all *p* > 0.05, Bonferroni corrected). Similarly, in the PCG, no significant differences were found across the three time points (all *p* > 0.05, Bonferroni corrected).

### 3.8 Psychological resilience (CD-RISC)

**Figure 7:**
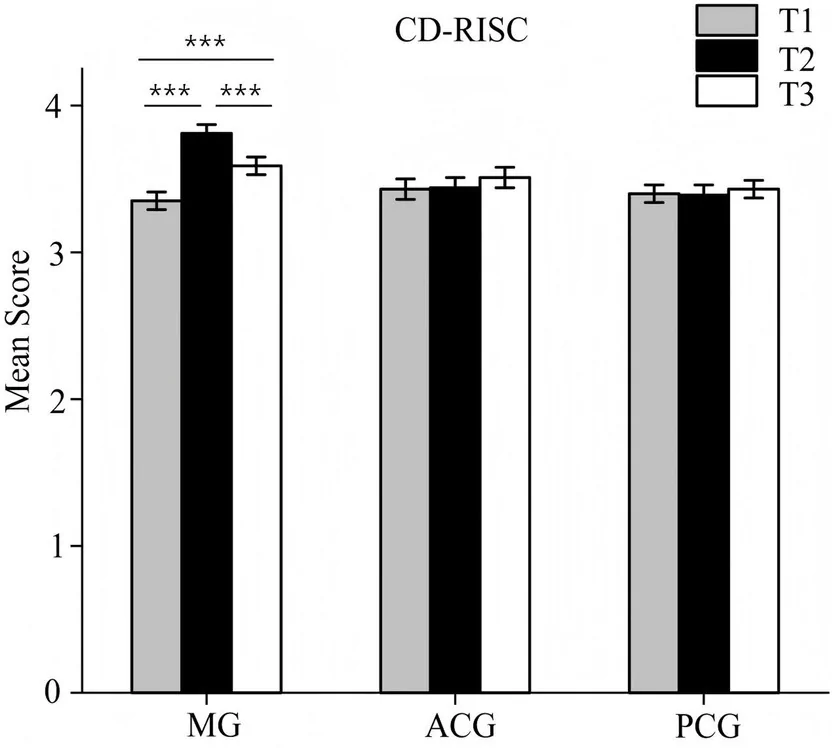
CD-RISC scores across groups and time points. Mean CD-RISC scores are presented for MG, ACG, and PCG at T1 (pretest), T2 (posttest), and T3 (follow-up). Error bars represent standard errors of the mean (SEM). Significant within-group differences between time points are indicated by asterisks. *p < 0.05; **p < 0.01; ***p < 0.001, Bonferroni-corrected.

As shown in Figure 7, a significant Group × Time interaction was observed for psychological resilience, *F* (4, 354) = 19.830, *p* < 0.001, *η*^2^*_p_* = 0.183 (Figure 7). Simple effect analysis indicated that in the MG, the resilience score at T2 was significantly higher than those at T1 (*p* < 0.001, Bonferroni corrected) and T3 (*p* < 0.001, Bonferroni corrected). The score at T3 was significantly higher than that at T1 (*p* < 0.001, Bonferroni corrected). In the ACG, no significant differences in resilience scores were observed between any of the time points (all *p* > 0.05, Bonferroni corrected). Similarly, in the PCG, no significant differences were found across the three time points (all *p* > 0.05, Bonferroni corrected).

A summary of the within-group changes across all outcomes is presented in Table 3.

**Table 3:** Within-group changes in outcome measures and between-group comparisons between the MG and ACG.

Outcome	MG: T2 − T1	ACG: T2 − T1	PCG: T2 − T1	MG: T3 − T1	MG vs. ACG at T2	MG vs. ACG at T3
	Δ*M*	Δ*M*	Δ*M*	Δ*M*	MD	MD
SMS	↑0.573^***^	↑0.013	↑0.048	↑0.201^**^	+0.471^***^	−0.033
FFMQ-SF	↑10.417^***^	↑0.783	↑0.567	↑1.883	+11.150^***^	+2.533
SAS	↓6.917^***^	↓1.750	↓1.896	↓0.708	−7.542^***^	−4.104
SDS	↓7.229^***^	↓1.271	↓0.333	↓1.438	−7.542^***^	−3.625
ACS	↑8.067^***^	↑2.450^*^	↑0.567	↑2.250^*^	+4.700^*^	+0.050
EIS	↑0.581^***^	↑0.097	↑0.031	↑0.248^**^	+0.387^**^	−0.015
CD-RISC	↑0.455^***^	↑0.011	↓0.011	↑0.235^***^	+0.369^**^	+0.075

MG, mindfulness training group; ACG, breathing-relaxation active control group; PCG, passive control group; ΔM, within-group mean change; MD, between-group mean difference. Within-group changes were calculated as T2 − T1 or T3 − T1. Upward and downward arrows indicate increases and decreases, respectively. Between-group differences were calculated as MG minus ACG. *p < 0.05; **p < 0.01; ***p < 0.001, Bonferroni-corrected.

## 4 Discussion

This study examined changes in seven psychological outcomes following a single 15 min mindfulness session, using both breathing-relaxation and no-intervention control conditions. Significant Group × Time interactions were observed across all outcomes. Compared with the ACG, the MG showed greater immediate improvements in state and trait mindfulness, attentional control, emotional intelligence, and resilience, together with lower SAS and SDS scores. At the two-week follow-up, however, none of the direct MG–ACG differences remained statistically significant. Within the MG, state mindfulness, attentional control, emotional intelligence, and resilience remained higher than baseline, whereas trait mindfulness, anxiety, and depressive symptoms did not differ significantly from baseline. These findings indicate that the intervention was primarily associated with immediate and selective short-term changes rather than stable or broadly sustained effects.

The immediate differences between the MG and ACG are particularly informative because the two conditions were matched in session duration, delivery format, researcher contact, and breathing practice. Unlike breathing relaxation, however, the mindfulness session additionally involved monitoring present-moment experience, recognizing mind wandering, redirecting attention, and adopting a non-judgmental attitude. These components may reduce cognitive–affective interference and support emotion regulation through attentional monitoring and acceptance, thereby producing broader immediate changes than physiological relaxation alone (Cásedas et al., 2024; Raugh et al., 2025). Consistent with this interpretation, attentional control was the only outcome that improved significantly within the ACG, whereas the MG showed changes across all seven outcomes. Attentional control may therefore represent one process through which mindfulness-specific components influence broader psychological functioning (Wang et al., 2024). Nevertheless, because attentional or emotional mechanisms were not directly tested, this interpretation remains tentative.

The outcomes of the SMS showed that the MG reported large gains in state mindfulness especially immediate after intervention. The state mindfulness is the ability to keep the focus on the current moment, and the great increase of the SMS scores will indicate that the intervention resulted in a significant immediate change in participants’ self-reported state mindfulness. The results align with the existing studies that point to mindfulness as the tool to enhance present-oriented attention and minimize the vulnerability to distraction by irrelevant thoughts or stressors (Wang et al., 2023). Gains in state mindfulness can be enhanced in training without necessarily evolving into a stable disposition, especially in lower-dose forms where engagement in the post-program reduces (Borgdorf et al., 2025; Buric et al., 2025). FFMQ scores need to be interpreted with care since mindfulness training is likely to change the way the participants react to the items in the long run. To prevent the over-interpretation of total score changes, it is important to investigate facet-level changes and measurement stability (Frances Levin-Aspenson et al., 2023).

These findings should also be interpreted in relation to the stability of the outcomes. State mindfulness is relatively context-sensitive, whereas trait mindfulness and broader psychological resources such as emotional intelligence and resilience are more stable characteristics. Therefore, a single 15 min session may produce clearer immediate changes in state-like outcomes than in enduring trait-like characteristics, while the immediate SAS and SDS changes should be interpreted cautiously because both scales assess symptoms over the preceding week.

SAS and SDS scores were lower immediately after the mindfulness session. However, because both scales assess symptoms over the preceding week, the T1 and T2 reference periods substantially overlapped. These immediate differences should therefore be interpreted as possible shifts in self-reported affect or symptom appraisal rather than as definitive reductions in past-week anxiety and depressive symptoms. The active control (ACG) and passive control (PCG) groups, in turn, exhibited relatively weak changes, which implied that the effects in the MG could be explained by the mindfulness intervention, but not by such factors as time or expectancy (Alkan et al., 2025). Such findings can be compared to the previous studies that indicate that mindfulness-based programs are effective in reducing anxiety and depression through lowering emotional reactivity and improving emotional regulation (Hoge et al., 2022). Previous findings indicate that mindfulness training reduced emotional dysregulation, including maladaptive rumination and increased stress reactivity (Horwitz et al., 2024). From a clinical application perspective, the significant reductions in SAS and SDS scores suggest that mindfulness may serve as a highly feasible and accessible intervention for improving emotional symptoms among non-clinical populations or students at risk, especially in the case of acute emotional distress.

The results from ACS, EIS, and CD-RISC support the view that mindfulness enhances an individual’s psychological function by synergistically improving cognitive control, emotion regulation, and adaptive coping abilities, thereby promoting a more stable and resilient mental state. The MG showed large improvements in attentional control as compared to the ACG and PCG. This observation is consistent with emerging literature that attentional control is one of the central processes underlying MBIs (Salem et al., 2025; Wang et al., 2024). Mindfulness training can make one less prone to distraction by negative or stress-inducing thoughts, by enhancing the capacity to attend to present-moment experience (Hoge et al., 2013). In the current research, the increased attentional control might have led to the decreased anxiety and depression through the reduction of the cognitive load related to the ruminative and intrusive thoughts (Kim et al., 2025). Meanwhile, mindfulness training increased emotional awareness and regulation, which are the primary elements of EI. This result is consistent with the existing research which has shown that mindfulness enhances emotional regulation which is one of the main features of EI (Lin et al., 2025). Mindfulness has been reported to positively affect EI and general psychological performance as it improves emotion control measures and communication effectiveness (Martínez-Pérez et al., 2023). The capacity to overcome adversity, resilience, also enhanced significantly in the MG. These results are in line with the past studies that have shown that mindfulness is an effective method to cultivate resilience through adaptive coping and emotional reactivity (Fortes et al., 2025). It has been demonstrated that mindfulness programs can improve resilience by improving emotional control and attentional control, which are essential in stress and adversity management (Lin et al., 2025). The identified improvements are indicative of the fact that mindfulness training does not only reduce emotional distress, but also develops psychological resources that can be used to cope with life challenges more efficiently.

Finally, we note that the differences between ACG and PCG were relatively small, indicating that different control conditions had a limited impact on the overall outcome pattern. In other words, neither breathing relaxation nor passive waiting produced changes comparable to those in MG, further supporting the explanation that improvements in MG are more likely due to the specific effects of mindfulness training itself, rather than the control method, time effect, or expectation effect.

### 4.1 Limitations and future directions

There are a number of limitations that should be considered. First, the participants were drawn from a non-clinical sample of university students within a relatively narrow age range, which limits the generalizability of the findings to clinical populations, older adults, and non-student groups. Even though having active and passive control groups strengthens causal inference, it is still impossible to exclude nonspecific factors such as expectancy effects or differences in engagement. Future research would benefit from more closely matched active control conditions to better isolate the specific components of the intervention (Hoge et al., 2022; Horwitz et al., 2024). In addition, both the SAS and SDS assess symptoms experienced during the preceding week. Because T2 was administered immediately after the intervention, its reference period substantially overlapped with that of T1, limiting the extent to which immediate score differences can be interpreted as actual changes in past-week anxiety and depressive symptoms. Future studies should therefore include measures that are more sensitive to immediate emotional changes. The follow-up duration was confined to three points of assessment and further longitudinal information is required to confirm whether the observed improvements are maintained, reduce, or further develop over the extended periods (Linardon et al., 2024; Wang et al., 2023). Lastly, a formal examination of attentional control as a mediator in the current analyses was not conducted. Preregistered longitudinal mediation models and multi-method assessments, such as behavioral or neurocognitive measures, should be used in the future in order to assess the proposed mechanisms more directly (Gupta et al., 2025).

## 5 Conclusion

Mindfulness training had larger post-intervention outcomes in anxiety and depressive symptoms, as well as simultaneous changes in mindfulness awareness, attentional control, emotional intelligence, and psychological resilience compared to both active and passive control groups. These results contribute to the current understanding of the short-term effects of brief mindfulness training on psychological functioning and suggest that sustained benefits may require continued practice or longer intervention periods.

## Data Availability

The datasets presented in this study can be found in online repositories. The names of the repository/repositories and accession number(s) can be found below: OSF: https://osf.io/uhj5v/.
